# Effects of cultural burning on insect-associated nematodes in the soil

**DOI:** 10.17912/micropub.biology.002263

**Published:** 2026-08-15

**Authors:** Chloe L. Golde, Yuerong Xiao, Charlene Nijmeh, Joseph Torres, Ron W. Goode, Jesse Valdez, Christina Oraftik, Yeva Allyn, Oujen T. Bortai, Natalie De La Cruz, Alyxx A. Ford, Chloe M. Hannsz, Julia N. Jackson, Abigail H. M. Kim, Z. Niobe Nabahe, Kala'i A. Romanchak, Osiris de Jesus Romero-Caldera, Siena J. Settle, Rahm Sheinfeld, Ava K. Stafford, Kyra L. Standing Crow, Cornelia M. T. D. Tiger, Amaury Payelleville, Tadashi Fukami

**Affiliations:** 1 Department of Biology, Stanford University, Stanford, CA, United States; 2 Department of Earth System Science, Stanford University, Stanford, CA, United States; 3 Muwekma Ohlone Tribe of the San Francisco Bay Area; 4 North Fork Mono Tribe; 5 Consultant to North Fork Mono Tribe; 6 BIO/NATIVEAM 35N Traditional Ecological Knowledge for Land Stewardship, Stanford University, Stanford, CA, United States; 7 DGIMI, Université de Montpellier, Montpellier, 34090, France

## Abstract

The Muwekma Ohlone Tribe of the San Francisco Bay Area conducted a cultural burn in oak woodland as part of cultural revitalization efforts. This low-intensity burn only moderately affected soil nematodes that infest acorn-feeding insects. The nematodes were frequently found after the burn, even within burned piles. This finding suggests that cultural burning may help to prevent outbreaks of acorn-feeding insects not only directly by killing them with heat, but also indirectly by having only minor effects on the soil nematodes. Preventing outbreaks of acorn-feeding insects increases the likelihood of more acorns being available for wildlife and cultural use.

**Figure 1. Cultural burning, soil sampling, and nematode emergence:** (A) One of the 13 piles burned at the cultural burn at 'Ootchamin 'Ooyakma on the Muwekma Ohlone Tribal land on January 23, 2026 (photo: Marina Luccioni). (B) Mixing of ash with topsoil at one of the nine piles, where the mixing was conducted after the burning was completed (photo: Marina Luccioni). (C) Students collecting soil samples from an unmixed burned piles at the edge location three days after the cultural burn (photo: Tadashi Fukami) (D) Frequency of nematode emergence from White traps (see methods section for explanation) of soil samples from varying burn treatments (n = 41, 52, 35, 44, 41, and 37 traps for center/mixed, center/unmixed, edge/mixed, edge/unmixed, out/mixed, and out/unmixed, respectively). ns = p > 0.05.

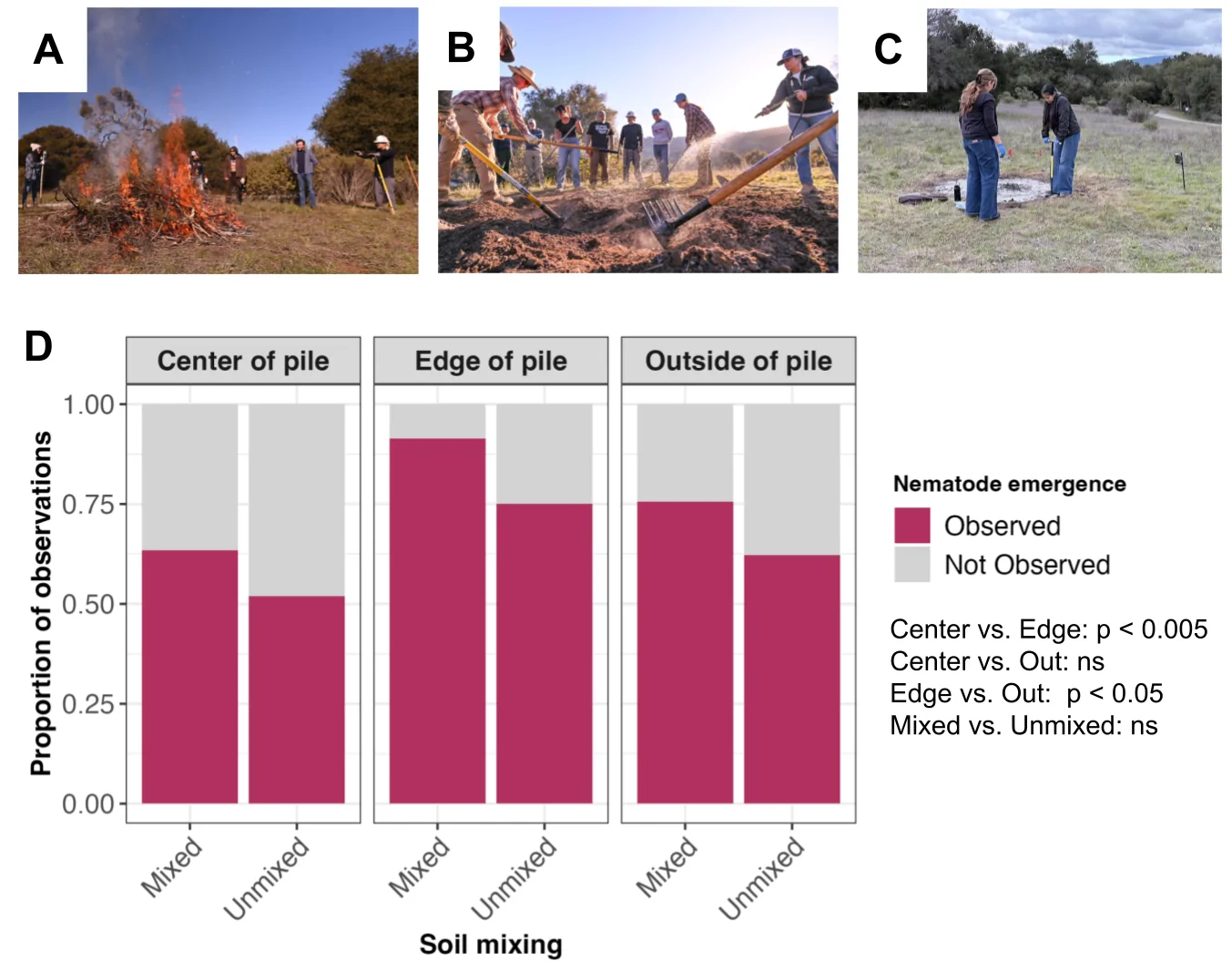



## Description

For thousands of years, Indigenous peoples around the world have practiced cultural burning (Hankins, 2024), or the “purposeful use of fire by a cultural group (e.g., family unit, Tribe, clan/moiety, society) for a variety of purposes and outcomes” (Clark et al., 2021). One of the many purposes of cultural burning in what is now called California is to maintain high production and quality of acorns as food and medicine (Long et al., 2016; Goode et al., 2023). In this region, cultural burning, often conducted in oak woodland before the gathering of new acorn crops, reduces the number of insects that feed on acorns, thereby keeping acorns available to people and other animals (Anderson, 2005; Halpern et al., 2022).

Two species of acorn-feeding insects are common and widespread in California, the filbertworm moth *Cydia latiferreana* and the filbert weevil *Curculio occidentalis* (Lewis, 1992). Adults of these insects lay eggs in fresh acorns, with larvae developing inside the acorn while it is still attached to the tree (Lewis, 1992). Once acorns drop, larvae emerge and burrow into the soil. During this process, larvae damage acorns, often making them unable to germinate and resulting in poor oak recruitment (Perea et al., 2020), as well as making them generally unsuitable for consumption by birds, mammals (including humans), and other animals, although the suitability of insect-infected acorns may vary among animal species. Cultural burning affects these insect life stages, with smoke possibly acting as a fumigant, reducing insect pool and limiting subsequent insect infestations.

In addition to fire, insect-parasitic organisms found in the soil, such as *Beauveria* and *Metarhizium* fungi and *Steinernema* nematodes, can reduce the prevalence of acorn-feeding insects by infecting larvae (Bruck and Walton, 2007; Payelleville et al., 2026). While unable to kill insect larvae themselves, some other species of nematodes can also enter and reproduce in dead insect larvae (Payelleville et al., 2026). Thus, cultural burning may alter the abundance of acorn-feeding insects not just directly with heat and ash, but also indirectly by affecting soil-dwelling fungi and nematodes that use the insects for growth and reproduction. However, the extent to which cultural burning changes the abundance of these organisms is not well documented.

To address this question, an undergraduate class at Stanford University worked with members of the Muwekma Ohlone Tribe of the San Francisco Bay Area and the North Fork Mono Tribe to examine how cultural burning in the Muwekma’s ancestral homeland might influence the abundance of insect-feeding nematodes in the soil ( Figure 1A, 1B). For this purpose, the students collected soil samples from six of the 13 burned piles on January 27, 2026, three days after the burn ( Figure 1C ). Since the larvae of the acorn-feeding insects were difficult to obtain in adequate numbers for experiments, we used a more readily available insect, the greater wax moth *Galleria mellonella* , to study the abundance of soil-dwelling nematodes that use insects. Previous work on nematodes and fungi indicates that this moth is an informative substitute for the filbert worm moth and the filbert weevil (Payelleville et al., 2026). We focus on nematodes in this study as we found no fungus capable of killing insects in the soil samples.

Results ( Figure 1D ) indicate that soil samples collected from the edge of the burned piles had more nematode emergence from the insect larvae, compared to soil samples collected at the center of the piles (z = 3.4, p < 0.005) and from outside the piles (z = 2.5, p < 0.05). However, nematode emergence was frequently observed (from more than 50% of dead larvae) even in the center of the piles. The frequency of nematode emergence did not differ significantly between soil samples collected from the center of the piles and those from outside the piles (z = -0.93, p = 0.62). One possible reason for this nonsignificant result is that nematodes were disturbed by people walking and compacting the soil. Soil compression can compromise nematode movement (Kapranas et al., 2017). The soil outside the piles had about 50 people walking over it during the burning. In contrast, the soil collected at the edge of the piles was avoided when walking, which may have acted as a buffer for some nematodes.

Another aspect of cultural burning that we examined was how the process of mixing ash with topsoil after the burn affected insect-feeding nematodes. Following the Traditional Ecological Knowledge held by the North Fork Mono Tribe (Goode et al., 2023), whom the Muwekma Ohlone Tribe invited to their cultural burn, Chairman Goode of the North Fork Mono Tribe instructed participants to mix ash with topsoil once burning was complete ( Figure 1B ). The purpose of this mixing is to re-incorporate nutrients and encourage the growth of culturally important plants in the piles. However, the Chairman also had participants leave ash and charcoal intact without mixing in some of the piles for animals. Deer and small mammals have been observed to roll, paw, and dust-bathe in these materials to remove fleas, ticks, and mites (Goode et al., 2023). The soil mixing appeared to increase the frequency of nematode emergence, although this trend was not statistically significant (χ² = 1.15, df = 1, *p* = 0.28).

Overall, despite differences in prevalence, nematodes were commonly observed in all soil sample groups ( Figure 1D ). The heat from the fire and the subsequent exposure to ash may be the likely causes of reduction in nematodes, but fire did not decimate them. In contrast to intense fires, such as wildfires and non-Indigenous prescribed burning, this finding suggests that cultural burning may have only modest effects on soil nematodes that infect insect larvae. Although this study focused on pile burning, the effects we observed in this study may extend to other types of cultural burning, such as broadcast burning, which similarly applies low-intensity fire to surface soil. Such fire is likely to allow soil-borne insect pathogens to recover quickly following the burn, maintaining their role in regulating insect populations.

Observations in this study were limited to immediate post-burn responses. We plan to continue monitoring for a year to capture potential delayed effects of fire on soil nematodes and fungi. Furthermore, in this study, we only confirmed the presence of nematode emergence. In the future, we plan to use a molecular sequencing approach (Payelleville et al., 2026) to obtain higher-resolution taxonomic data to understand how cultural burning may influence the composition of soil nematodes and fungi.

## Methods

Study site

This study was conducted at 'Ootchamin 'Ooyakma (Jasper Ridge Biological Preserve) in the eastern foothills of the Santa Cruz Mountains in California, on the ancestral homeland of the Muwekma Ohlone Tribe of the San Francisco Bay Area (Severson et al., 2022). A cultural burn was carried out on January 23–24, 2026, in an oak woodland habitat (Adami, 2026). The burn was led by Chairwoman Charlene Nijmeh of the Muwekma Ohlone Tribe and Chairman Ron Goode of the North Fork Mono Tribe, who was invited by the Muwekma Ohlone Tribe as their advisor for revitalization of cultural fire. In addition, the burn was conducted as collaborative work with Stanford researchers, students, preserve docents, and community members.

The burn was guided by Indigenous intentions to care for oak woodland and restore relationships between fire, plants, animals, and people. Burn piles consisted of woody material on site ( Figure 1A ; see also the video and photos in Adami, 2026), including freshly cut branches of coast live oak ( *Quercus agrifolia* ) and coyote brush ( *Baccharis pilularis* ). No branches thicker than an average adult person’s arm were added to the piles. Piles were burned the same day they were made. Fire was started with a lighter, and no drip torch was used. Post-burn treatments were differentiated based on ash and charcoal handling (Goode et al., 2023). In nine of the 13 burned piles (mixed piles), ash and charcoal deposits were intentionally incorporated into the upper soil layer by raking with garden rakes ( Figure 1B ). In the remaining four piles (unmixed piles), these materials were left on the soil surface so deer and other animals can use the piles for ash bathing (Goode et al., 2023).

Soil collection

Soil was collected post burn by the Stanford BIO/NATIVEAM 35N students on January 27, 2026 ( Figure 1C ). About 1 kg of soil was collected from each of six burn piles, three mixed and three unmixed. Samples were collected in the center of the burn site (“center”), the edge of the ashes (“edge”), and 2 to 3 meters away from the end of the burn site (“out”) for each of the six piles, resulting in a total of 18 sampling locations. Each sample consisted of three soil cores extracted by a bulb planter, each of 10 cm diameter and a depth of 15 cm from the surface. For center/unmixed samples, the layer of ash on the surface was crudely brushed away before sampling. Soil samples were filtered through a 3.2-cm sieve to remove large rocks and debris, then placed in new plastic bags for about 30 minutes for transportation to the laboratory.

Bait trapping

On the same day as soil sampling, 20 *G. mellonella* larvae of roughly the same size and life stage (Carolina Biological Supply), were added to each of the 18 bait traps. Each trap consisted of the sieved soil from one of the 18 sampling locations in a glass container (20.3 cm long, 15.2 cm wide, and 5.1 cm deep) sealed with a plastic lid. Small holes were poked around the lid. The traps were stored at room temperature (20–26°C). Each subsequent week, the students checked for larval death by dumping the soil into an aluminum bin and picking out each larva. Each dead larva was added to a separate “modified White trap” (Payelleville et al., 2026). Live larvae were mixed into the soil and returned to the bait trap. This procedure was repeated weekly until March 3, 2026, when all larvae were either dead or unable to be found.

White trapping

Modified White traps (White, 1927) were made as described in Payelleville et al. (2026), an entomopathogen study conducted at 'Ootchamin 'Ooyakma. These traps allowed us to check whether any nematode entered and multiplied in the dead insect larva and then emerged from the cadaver. The White traps contained approximately 20 ml of Ringer’s solution to moisten the filter on which the insect cadaver was deposited. White traps were stored at room temperature (20-26 °C). All traps were checked for nematode emergence under a dissection microscope on March 13, 2026. Nematode emergence was determined by visible presence of nematodes in ringer solution. Common entomopathogenic fungi of the genera *Beauveria* and *Metarhizium* were also visually checked for under the microscope, but none was observed.

Statistical analysis

The presence/absence of nematode emergence from the insect cadavers was analyzed in a binomial generalized linear mixed model, with soil sampling location (center, edge, or out), mixing of ash with topsoil (mixed or unmixed), and their interaction term as fixed factor and pile identity as a random factor.

Classroom pedagogy

This study was conducted over seven weekly laboratory sessions as part of the BIO/NATIVEAM 35N: Traditional Ecological Knowledge (TEK) for Land Stewardship class at Stanford University. To gain basic knowledge of insect-feeding nematodes, students first read Payelleville et al. (2026) in a seminar-style session. Then, in the first laboratory session, students participated in the soil sampling and established bait raps with the collected soil. In the following sessions, students checked for larvae in the bait traps and made White traps with each dead insect. In the last session, they checked if nematodes were found in White traps and discussed the results. Throughout the seven weeks, the students also learned about TEK by listening to Indigenous scholars and discussing TEK-related literature, with an emphasis on cultural burning in California. All students from the class are co-authors of the paper.
